# Valorisation of Edible Mushroom By-Products as Natural Flavour Enhancers in Plant-Based Foods: Mechanisms, Sensory Profiles, and Industrial Translation

**DOI:** 10.3390/foods15183255

**Published:** 2026-09-15

**Authors:** Hadeel Edkaidek, Divakar Dahiya, Poonam Singh Nigam

**Affiliations:** 1 Department of Food Processing, Faculty of Agriculture, Palestine Technical University-Al-Aroub Branch, Hebron, Palestine; 2 Basingstoke and North Hampshire Hospital, Basingstoke RG24 9NA, UK; 3 Biomedical Sciences Research Institute, Ulster University, Coleraine BT52 1SA, UK

**Keywords:** plant-based foods, mushroom, by-products, Umami, flavour enhancement, sensory evaluation, circular economy

## Abstract

Plant-based foods have emerged as an important choice for many consumers. Although foods formulated from plant-derived raw materials and ingredients can contribute to more sustainable food systems, their broader consumer acceptance continues to be constrained by suboptimal flavour quality compared with products prepared using animal-derived ingredients. While considerable research has focused on improving the texture, nutritional value, and processing of plant-based foods, recreating the savoury depth characteristic of animal-derived foods remains a persistent challenge. Edible mushroom by-products have attracted increasing attention as sustainable sources of flavour-enhancing compounds; however, their functionality cannot be explained solely by the presence of glutamate or other individual constituents. Rather, their sensory effects arise from coordinated interactions among umami-active molecules, receptor-mediated taste mechanisms, food matrix properties, and human perception. This review examines the potential of edible mushroom by-products as natural flavour-enhancing ingredients for plant-based foods. It also reviews evidence on flavour-active composition, the molecular basis of umami perception, glutamate–5′-nucleotide synergism, formulation strategies, sensory evaluation, consumer acceptance, and industrial translation.

## 1. Introduction

The transition toward more sustainable food systems has accelerated interest in plant-based foods as demand grows for diets with lower environmental impacts [1]. Compared with livestock production, plant-based dietary patterns are generally associated with reduced greenhouse gas emissions, lower resource use, and favourable public health outcomes, making them an important component of future food systems [2,3]. Beyond their environmental advantages, well-formulated plant-based foods may contribute to health-promoting dietary patterns when they provide nutritionally appropriate alternatives to animal-derived foods. However, these potential benefits depend on the nutritional quality and formulation of the final product rather than on its plant-based origin alone [3].

Nevertheless, broader consumer adoption remains constrained by factors extending beyond sustainability [4]. While environmental and health considerations often motivate initial product trial, repeated consumption depends largely on eating quality, with flavour consistently identified as one of the principal determinants of consumer acceptance [4,5,6].

Flavour remains one of the greatest challenges in plant-based food development. Many plant protein ingredients provide limited savoury perception while contributing beany, grassy, bitter, or astringent off-flavours that reduce overall palatability [7]. Although advances in ingredient functionality and product structuring have substantially improved texture and appearance, reproducing the sensory richness associated with animal-derived foods remains difficult [7,8]. Consequently, growing attention has focused on naturally derived flavour-enhancing ingredients capable of improving sensory quality while supporting clean-label product development [9].

In parallel, circular economy strategies have stimulated interest in valorising agro-industrial by-products as functional food ingredients [10]. Among these, by-products generated during the cultivation, grading, trimming, and processing of edible mushrooms are of particular interest because several fractions retain compounds with potential nutritional and technological value despite being underutilised [11]. In this review, mushroom by-products specifically refer to secondary material streams generated during the cultivation, harvesting, grading, trimming, or processing of edible mushrooms that are not retained as the primary commercial mushroom product [10,11]. Examples reported in the literature include misshapen or off-grade mushrooms, stems or stipes, and other mushroom processing residues [11]. Spent mushroom substrate is considered separately because it represents a residual stream from mushroom cultivation rather than an edible mushroom fraction. Mushroom powders, extracts, and hydrolysates are not considered by-products per se; they are classified as by-product-derived ingredients only when their starting material is explicitly identified as a mushroom by-product and has subsequently been processed into an ingredient [12,13]. This distinction is important because the composition and processing history of these materials can influence their flavour active constituents and functionality in food formulations.

Mushroom by-products may contain diverse flavour-active constituents, including free amino acids, peptides, nucleotides, soluble sugars, and volatile compounds, although their composition varies according to mushroom species, tissue type, cultivation conditions, and subsequent processing [11]. Their potential as flavour enhancers should not, however, be attributed solely to the presence of glutamate or other individual umami compounds [14]. Experimental evidence indicates that combinations of taste-active compounds can contribute to umami and savoury perception. For example, Guo et al. demonstrated that enzymatic hydrolysis of defective *Morchella sextelata* fruiting-body material generated fractions containing multiple umami-related contributors, including low-molecular-weight peptides, free amino acids, and 5′-nucleotides [12]. Similarly, sensory evaluation of *Schizophyllum* commune extract demonstrated umami-related sensory characteristics and an enhancement of taste perception when the extract was incorporated into salt solutions and seasoned clear soup [15]. These findings suggest that the flavour contribution of mushroom-derived materials may arise from the combined effects of multiple taste-active constituents and their interactions rather than from a single dominant compound.

Importantly, evidence obtained from extracts, hydrolysates, or model solutions cannot necessarily be extrapolated directly to complex plant-based food matrices. Processing conditions, ingredient concentration, matrix composition, and interactions among flavour-active compounds can modify flavour release and sensory perception [16,17]. Therefore, evaluating mushroom by-products as flavour enhancers requires consideration not only of their chemical composition but also of how their flavour functionality is expressed within the target food matrix.

Against this background, this review examines the valorisation of edible mushroom by-products as natural flavour enhancers in plant-based foods, with emphasis on the mechanisms underlying their flavour contribution, sensory profiles, and potential for industrial translation. The review considers the composition and flavour-active constituents of relevant mushroom by-product streams, the molecular basis of umami perception and taste interactions, and the effects of processing and food-matrix characteristics on flavour expression. It further evaluates reported applications in plant-based food formulations, available sensory and consumer evidence, and the technological and practical limitations that may constrain their broader application. By integrating compositional, mechanistic, sensory, and application-oriented evidence, this review aims to clarify not only the potential of mushroom by-products as natural flavour enhancers but also the conditions and limitations that determine their effectiveness in plant-based food systems.

A focused literature search was conducted using Google Scholar to identify peer-reviewed publications relevant to the scope of this narrative review. Searches combined terms related to mushroom by-products, edible mushrooms, flavour-active compounds, umami, sensory evaluation, plant-based foods, off-flavour masking, and industrial food applications. Particular emphasis was placed on literature published from 2020 onward, while earlier publications were considered only when necessary to provide essential scientific background. Priority was given to recent original research articles reporting experimental findings and relevant review articles providing broader scientific context. Publications that were not directly related to the sensory, compositional, mechanistic, food-application, or industrial aspects addressed in this review were excluded from consideration.

## 2. Valorisation Potential of Residual Mushroom Biomass

Mushroom cultivation and commercial processing generate several residual biomass streams, including stipes, processing cuttings, broken fruiting bodies, and mushrooms that do not meet commercial specifications [18]. The scale of these streams is substantial: approximately 20% of fresh mushroom weight may be discarded during production, with stipes, broken fruiting bodies, and non-compliant products representing important fractions [18]. However, these materials are not chemically uniform. Their composition varies with mushroom species, tissue type, and the stage at which the material is separated from the commercial product [18,19].

The composition of residual fractions provides a basis for their valorisation as food ingredients. Bermúdez-Gómez et al. [19] reported that flour obtained from *Agaricus bisporus* stem coproducts contained approximately 14 g/100 g protein, whereas *Pleurotus ostreatus* stem coproducts contained 36.62–40.34 g/100 g β-glucans and 20.57–39.86 g/100 g linoleic acid on a lipid basis. Both mushroom species also contained free amino acids and showed umami-related characteristics [19]. Similarly, Prandi et al. (2023) found that mushroom processing residues contained proteins, amino acids, and other potentially recoverable components, although their concentrations differed among *A. bisporus, Lentinula edodes*, and *P. ostreatus* [18]. These compositional differences are particularly relevant to flavour-oriented valorisation because the sensory functionality of a residual fraction depends not only on the total amount of nutrients or bioactive compounds but also on the presence and accessibility of specific taste-active constituents [14].

Post-harvest handling can substantially modify this composition and, consequently, the flavour potential of the resulting ingredient. Fresh mushroom residues contain more than 80% water, creating conditions that favour rapid quality deterioration and requiring stabilisation before further processing [19]. In a study of mushroom by-products from *A. bisporus, L. edodes,* and *P. ostreatus*, Prandi et al. dried the residues at 45–55 °C to reduce their moisture content to approximately 10% before extraction [18]. This illustrates that drying is not merely a preservation step but can also become a critical processing variable that determines the chemical state of the material entering subsequent valorisation processes.

Direct evidence for this effect has been reported for *L. edodes*. Zhang et al. compared freeze-drying, hot-air drying, and natural drying and observed substantial changes in both volatile and non-volatile flavour compounds. Hot-air-dried mushrooms contained 56.55 μg/g cyclic sulfur compounds, compared with 7.24 μg/g in fresh mushrooms, 3.90 μg/g after freeze-drying, and 0.04 μg/g after natural drying. In contrast, freeze-dried samples exhibited the highest equivalent umami concentration, reaching 29.88 g MSG equivalents/100 g. The authors also reported that drying increased 5′-nucleotide levels while reducing organic acids and free amino acids. Thus, drying may shift the balance between aroma-active and taste-active constituents rather than producing a uniform increase or decrease in flavour intensity [20]. This distinction is important when residual biomass is intended for use as a flavour ingredient, because the processing conditions that favour a desirable aroma profile may differ from those that maximise umami-related characteristics.

Drying temperature provides a further example of this process dependence. Hou et al. evaluated hot-air drying of *Suillus granulatus* at 40, 50, 60, 70, and 80 °C. The material dried at 60 °C showed significantly higher equivalent umami concentration, taste activity values for glutamic acid and 5′-GMP, and electronic-tongue umami scores than samples treated at the other temperatures. The study also identified 71 volatile compounds, including 16 key odour-active compounds, and showed that drying temperature altered the overall aroma profile [21]. These results indicate that drying conditions should be selected according to the intended sensory function of the ingredient rather than solely according to dehydration efficiency. A condition that preserves or promotes desirable volatile compounds may not necessarily produce the highest concentration or sensory expression of non-volatile taste-active compounds.

Enzymatic processing can provide an additional route for modifying the flavour functionality of residual mushroom material. Gao et al. used defective fruiting bodies of *Morchella sextelata* and compared different enzymatic systems for the production of umami-rich hydrolysates. The optimised Neutrase–Flavourzyme treatment achieved a degree of hydrolysis of 36.64% and generated hydrolysates containing 224.83 ± 0.87 mg/g free amino acids and 4.84 ± 0.32 mg/g 5′-nucleotides. Peptides in the 1–3 kDa range contributed more strongly to the umami and saltiness of the resulting hydrolysates [12]. This study demonstrates that enzymatic treatment can convert defective mushroom biomass into fractions with enhanced accessibility of taste-active compounds. However, the presence of abundant free amino acids or peptides should not be interpreted as direct evidence of superior flavour in a finished food, because sensory expression depends on the concentration and combination of taste-active compounds and on the surrounding food matrix. The relevance of hydrolysis therefore lies in its ability to modify the composition and accessibility of flavour-active fractions, rather than simply increasing total extractable solids.

Extraction technology represents another important determinant of the composition and functionality of recovered fractions. Prandi et al. [18] compared aqueous, ultrasound-assisted, and enzyme-assisted extraction of proteins from six mushroom by-product streams. Enzyme-assisted extraction produced the highest protein extraction yield, although the recovered fraction consisted predominantly of peptides rather than intact proteins. In contrast, aqueous extraction produced lower yields but generated purer protein fractions, whereas ultrasound-assisted extraction resulted in a lower degree of hydrolysis but relatively high racemization under the conditions tested [18]. These findings illustrate why extraction yield alone is insufficient for evaluating the valorisation potential of mushroom residues. The most efficient process in terms of total recovery may not generate the fraction with the most desirable composition or functionality for a specific food application. For flavour-oriented applications, selective recovery of amino acids, peptides, nucleotides, or other flavour-active compounds may therefore be more relevant than maximising total protein or solids recovery.

Residual mushroom biomass can also provide technological functionality in addition to its compositional value. Umaña et al. [22] evaluated a concentrate obtained from *A. bisporus* by-products as an emulsifier in oil-in-water emulsions. At 5 and 7.5% (*w*/*w*), the mushroom concentrate increased emulsion viscosity, promoted shear-thinning behaviour, reduced droplet migration, and improved stability against changes in droplet size. Although this study did not directly evaluate flavour enhancement, it demonstrates that mushroom residual streams may contribute simultaneously to ingredient functionality and formulation performance. Such multifunctionality could be advantageous in plant-based food systems, where a single upcycled ingredient may need to contribute to flavour while also interacting with the physical properties of the food matrix [22].

Overall, the available evidence indicates that the valorisation potential of residual mushroom biomass is strongly dependent on processing history. Species and tissue type determine the initial composition, whereas drying, temperature, enzymatic treatment, and extraction can modify the concentration, accessibility, and distribution of compounds relevant to flavour and technological functionality [12,18,20,21]. Residual mushroom biomass should therefore be viewed as a process-dependent raw material rather than a chemically uniform by-product. For the development of natural flavour enhancers, the key challenge is not simply to recover compounds from an underutilised stream, but to control processing conditions so that the resulting ingredient retains or selectively enriches the taste- and aroma-active constituents required for its intended plant-based food application.

**Figure 1:**
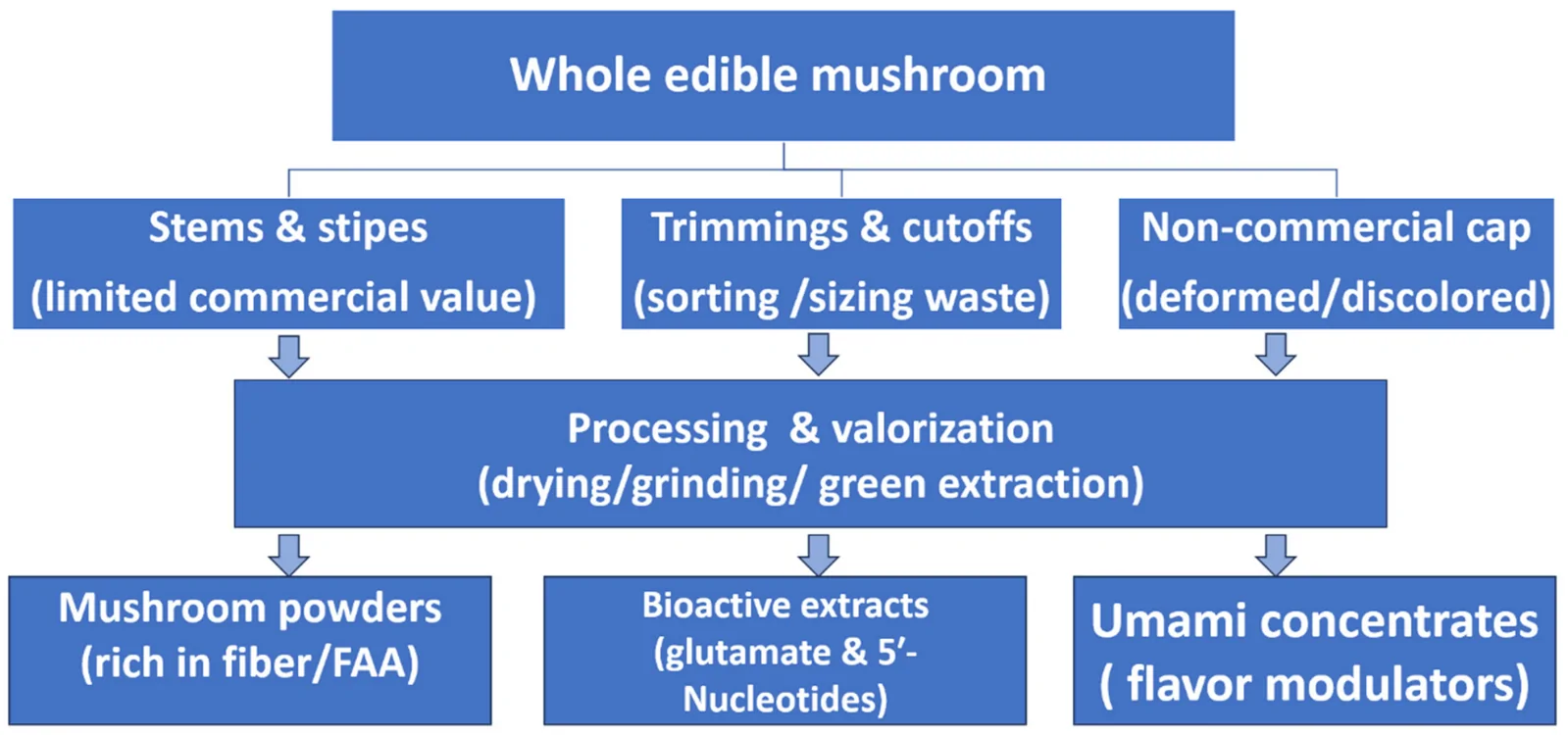
Schematic flowchart illustrating the valorisation pathway of edible mushroom fractions, commercial trimmings, and non-commercial residues into value-added functional by-products (powders, bioactive extracts, and umami flavour modulators) for food applications.

The overall conceptual framework for converting raw mushroom components, including stems, trimmings, and non-commercial portions, into novel bioactive and flavour-enhancing ingredients for food applications is schematically illustrated in Figure 1.

## 3. Flavour-Active Compounds in Mushrooms

Mushroom derived-materials can contribute to food flavour through both non-volatile taste-active compounds and volatile aroma-active compounds. Free amino acids, 5′-nucleotides, and peptides contribute primarily to taste, whereas volatile compounds contribute to aroma and influence flavour through retronasal olfaction [23]. The relative contribution of these compound classes varies among species and mushroom fractions, making glutamate concentration alone an insufficient indicator of flavour potential [14,24].

### 3.1. Taste-Active Compounds

Free amino acids and 5′-nucleotides are important contributors to mushroom taste. Glutamate and aspartate are associated with umami perception, while 5′-GMP and 5′-IMP can enhance umami when present with glutamate [24,25]. Their sensory relevance, however, depends on the combined profile of taste-active constituents rather than on glutamate concentration alone.

Experimental evidence illustrates this compositional complexity. In *Schizophyllum* commune extract, glutamic and aspartic acids occurred alongside other free amino acids and 5′-nucleotides, and the extract exhibited salty and umami taste characteristics [15]. Peptides constitute another class of potentially important taste-active compounds. Recent studies have identified umami-active peptides from edible mushrooms, including Agaricus bisporus, with measurable umami thresholds and interactions with the T1R1/T1R3 receptor system [26]. These findings indicate that mushroom flavour cannot be adequately represented by a single chemical marker, because different classes of taste-active compounds may contribute simultaneously to the perceived taste profile.

### 3.2. Aroma-Active Compounds

Taste alone does not account for the characteristic flavour of mushrooms. Volatile compounds contribute directly to aroma, with alcohols, ketones, aldehydes, and sulfur-containing compounds among the major classes reported in edible mushrooms [27]. In *Agaricus bisporus* and *Pleurotus ostreatus,* 1-octen-3-one, 1-octen-3-ol, and methional were identified among the key odourants, while cooking substantially altered the volatile profiles [28].

The aroma profile can also differ markedly according to processing conditions. In *Lentinula edodes*, drying altered eight-carbon volatiles and sulfur-containing compounds, while hot-air drying produced substantially higher concentrations of cyclic sulfur compounds than fresh, natural-dried, or freeze-dried samples [20]. Such changes demonstrate that aroma potential is not an intrinsic and fixed property of mushroom biomass but depends partly on how the material is processed.

Sensory evidence further supports the contribution of aroma to mushroom flavour. *Schizophyllum* commune extract was characterised by mushroom, meaty, musty, and bitter-aromatic notes in addition to salty and umami tastes [15]. Thus, the flavour contribution of mushroom-derived materials may result from the combined perception of taste and aroma rather than from umami enhancement alone.

### 3.3. Implications for Flavour Enhancement

The coexistence of taste-active and aroma-active compounds provides a more appropriate basis for evaluating mushroom materials as natural flavour enhancers. Their relative abundance differs among species and fractions, and the presence of a particular compound does not necessarily predict its contribution to the final sensory profile. This is particularly relevant when residual mushroom materials are considered for valorisation, because their chemical composition may differ from that of the primary commercial product [19].

Accordingly, flavour potential should be evaluated using a combination of chemical characterisation and sensory evidence rather than through single-compound measurements. Studies using mushroom extracts and powders have demonstrated measurable effects on taste in model food systems, including enhanced umami and saltiness [15,29]. However, evidence from isolated extracts or individual mushroom materials should not be assumed to predict performance in every plant-based food formulation [17]. The extent to which these flavour-active constituents translate into sensory benefits depends on the characteristics of the ingredient and the final product.

Overall, the evidence indicates that mushroom flavour is a multidimensional property arising from the interaction of taste-active and aroma-active compounds rather than from a single chemical marker (Table 1). For residual biomass, this distinction is particularly important because processing and fraction-specific composition may alter the balance among these contributors. Consequently, chemical profiling should be combined with sensory validation in relevant plant-based food matrices before claims of flavour-enhancing functionality are made.

Because many flavour-active compounds have been characterised in whole edible mushrooms rather than specifically in residual fractions, these studies are considered here as mechanistic and compositional evidence rather than direct evidence of by-product valorisation.

**Table 1:** Representative taste- and aroma-active constituents reported in edible mushrooms and their flavour relevance.

Mushroom Species	Material Studied	Representative Flavour-Active Constituents	Processing or Experimental Context	Principal Finding
*Agaricus bisporus*	Fruiting bodies	1-Octen-3-one, 1-octen-3-ol, methional and other volatiles	Raw, boiled and oven-cooked	Cooking substantially altered the volatile profile; key odourants included 1-octen-3-one and methional [28]
*Pleurotus ostreatus*	Fruiting bodies	1-Octen-3-ol, methional and other volatiles	Raw, boiled and oven-cooked	Processing modified volatile abundance and the relative contribution of key odourants [28]
*Lentinula edodes*	Fruiting bodies	Free amino acids, 5′-nucleotides, sulfur compounds and eight-carbon volatiles	Freeze-drying, hot-air drying and natural drying	Drying altered both non-volatile and volatile flavour compounds; hot-air drying increased cyclic sulfur compounds [20]
*Lentinula edodes*	Fruiting bodies	Free amino acids, organic acids and 5′-nucleotides	Hot-air drying	Drying time changed non-volatile taste compounds and equivalent umami concentration [30]
*Schizophyllum commune*	Aqueous extract	Glutamic acid, aspartic acid, 5′-AMP, 5′-GMP and other amino acids	Extraction and model food systems	Extract showed salty and umami tastes and mushroom, meaty and musty aroma attributes [15]
*Agaricus bisporus*	Hydrolysate-derived peptides	Umami-active peptides	Hydrolysis, peptide screening and sensory evaluation	Several identified peptides exhibited umami activity and enhanced umami in combination with MSG or IMP [26]

The studies are summarized in Table 1 qualitatively because differences in sample preparation, moisture basis, extraction procedures, processing conditions, and analytical methods prevent direct quantitative comparison across species.

## 4. Umami Perception

Umami is particularly relevant to the development of savoury plant-based foods because it contributes to savoury taste and palatability, while the broader perception of depth and continuity depends on interactions among umami and other flavour-active components. Its perception is primarily associated with the detection of L-glutamate and is strongly modulated by other taste-active compounds, particularly 5′-ribonucleotides such as inosine 5′-monophosphate (IMP) and guanosine 5′-monophosphate (GMP) [25,31]. This distinction is important when considering mushroom-derived ingredients, because their flavour contribution cannot be predicted solely from the concentration of glutamate. The composition of the ingredient, the relative abundance of taste-active compounds, and their interactions within the food matrix can collectively determine the resulting sensory response [14,24].

The relationship between chemical composition and perceived umami is therefore not linear. Food processing and matrix characteristics can modify the availability and perception of taste-active compounds, while interactions among glutamate, nucleotides, peptides, and other flavour constituents may alter the final sensory response [17,24]. For mushroom-derived ingredients, this means that a high concentration of an individual umami compound does not necessarily translate into a proportionally stronger sensory effect. Such matrix dependence is particularly relevant to plant-based foods, where protein ingredients, lipids, carbohydrates, salts, and processing conditions can influence flavour release and perception [17].

### 4.1. Molecular Basis of Umami Taste Receptors

Umami perception involves specialized taste receptors expressed in taste receptor cells [32]. The best-established receptor system is the T1R1/T1R3 heterodimer, a class C G protein-coupled receptor that responds to L-glutamate and is modulated by 5′-ribonucleotides [33,34]. Structural and molecular studies indicate that glutamate and nucleotides interact with distinct but functionally coupled regions of the receptor, providing a mechanistic basis for their synergistic effect [35].

Following receptor activation, signalling through G-protein-dependent pathways involving PLCβ2, intracellular Ca^2+^ mobilization, and TRPM5 contributes to depolarization of taste receptor cells and transmission of the taste signal [25]. Although additional glutamate-sensitive receptors, including mGluR1 and mGluR4, have been investigated, their contribution to human umami perception remains less firmly established than that of T1R1/T1R3 [32,34]. Accordingly, these receptors should not be presented as equivalent components of the glutamate–nucleotide synergistic mechanism [36].

**Figure 2:**
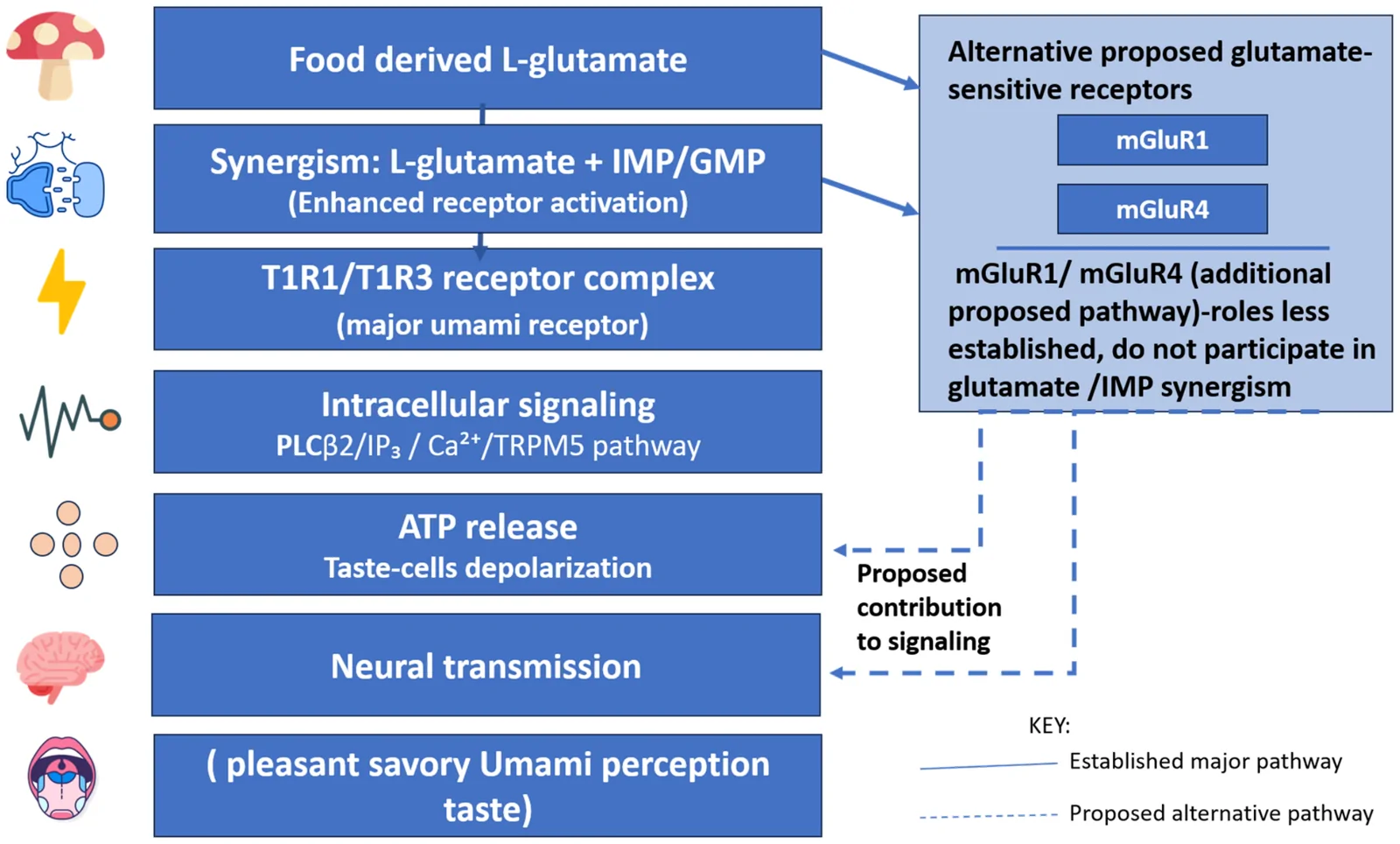
Proposed molecular mechanisms of umami taste perception and receptor-mediated signalling. (Drawn by first author).

The established and proposed molecular pathways underlying umami perception are summarised in Figure 2, highlighting the primary role of T1R1/T1R3 and the proposed contribution of additional glutamate-sensitive receptors.

Schematic overview of the established and proposed signalling pathways involved in umami taste perception. Food-derived L-glutamate primarily activates the T1R1/T1R3 heterodimer complex (established major pathway, solid arrows), a process markedly enhanced through synergism with purine nucleotides (IMP/GMP). Receptor activation triggers downstream intracellular signalling via the PLCβ2/IP_3_/Ca^2+^/TRPM5 pathway, inducing taste-cell depolarization, ATP release, and neural signal transmission to the brain. Alternative glutamate-sensitive receptors, including mGluR1 and mGluR4 (proposed pathways, dashed arrows), may provide additional contributions to umami signalling under specific physiological conditions, operating independently of the primary T1R1/T1R3 receptor complex.

### 4.2. Synergistic Interactions

The most established form of umami synergism involves L-glutamate and the 5′-ribonucleotides IMP and GMP [25,35]. Rather than acting independently, these compounds interact to enhance umami signalling and perception when present together. Molecular studies indicate that nucleotide binding can stabilise the active conformation of T1R1/T1R3 and increase the receptor’s responsiveness to glutamate [35].

This mechanism is particularly relevant to mushroom-based flavour enhancement because edible mushrooms can contain both umami-active amino acids and 5′-nucleotides. A comparative study of five edible mushroom species demonstrated substantial interspecies differences in the relative contribution of umami amino acids and 5′-nucleotides. *Pleurotus eryngii*, for example, showed a particularly high content of flavour-active 5′-nucleotides, whereas *Oudemansiella radicata* showed a greater contribution from umami amino acids [37]. These findings illustrate why mushroom species cannot be treated as chemically equivalent sources of umami and why the selection of a mushroom-derived ingredient should consider its specific taste-active profile rather than total glutamate content alone.

The sensory consequence of this interaction also depends on the food context. The same mushroom ingredient may produce different flavour effects according to its concentration, processing history, and the composition of the receiving matrix [17]. This is particularly important in plant-based foods, where mushroom-derived ingredients may interact with protein-associated off-flavours and other matrix components that influence flavour release and perception [7,38].

Evidence from food formulations supports this matrix-dependent interpretation. Incorporation of *Agaricus bisporus* into plant-based burgers has been associated with changes in physicochemical and sensory properties, while oyster mushroom mycelium has been reported to affect both physical characteristics and flavour properties of plant-based beef analogues [39,40]. These studies are relevant because they demonstrate that mushroom materials can modify the sensory profile of formulated foods, but they do not establish that umami receptor synergism alone accounts for the observed effects. Such effects may result from concurrent changes in taste-active compounds, volatile composition, texture, and matrix interactions.

Taken together, the evidence supports a more restrained interpretation of mushroom-derived umami enhancement. Glutamate–nucleotide interactions provide a well-established molecular mechanism, but the sensory contribution of mushroom ingredients in plant-based foods is likely to arise from the combined effects of taste-active compounds, aroma, matrix interactions, and processing. This distinction is important for industrial formulation because optimising mushroom ingredients requires evaluation in the final food matrix rather than relying exclusively on compositional measurements or isolated receptor assays.

## 5. Consumers’ Sensory Responses

The practical value of mushroom-derived ingredients is ultimately determined by their performance in the finished food rather than by their chemical composition alone. In plant-based foods, sensory quality results from the combined perception of taste, aroma, texture, and aftertaste, while processing and matrix composition can modify the expression of flavour-active constituents [8,17,41]. Accordingly, the sensory effect of a mushroom-derived ingredient should be interpreted as a matrix-dependent outcome rather than as an inherent property of the ingredient itself [8,17].

Available evidence supports the potential of mushroom-derived materials to modify sensory quality, but the response is not consistently positive across formulations or inclusion levels [39,40]. Studies using mushroom mycelium, powders, or other mushroom-derived materials have reported changes in savoury perception, aroma, bitterness, astringency, and overall acceptability, with the magnitude and direction of these effects depending on ingredient concentration and the surrounding food matrix [39,40]. Thus, the relevant question is not simply whether mushroom materials enhance flavour, but under which formulation and processing conditions their contribution improves the overall sensory balance.

A meaningful assessment of sensory improvement also requires comparison with the animal-derived foods that plant-based products are commonly designed to emulate. Animal-derived foods possess complex flavour profiles generated through interactions among amino acids, peptides, nucleotides, lipids, and volatile compounds formed during cooking, whereas plant-based analogues may exhibit lower levels or different distributions of desirable savoury and aroma compounds alongside characteristic beany, grassy, or other off-flavours [7,16,42]. Direct instrumental and sensory comparisons between commercial plant-based beef analogues and beef have demonstrated substantial differences in volatile composition and perceived aroma and taste following thermal processing [43]. This comparison provides an important benchmark for evaluating mushroom-derived ingredients: sensory improvement should not be interpreted simply as an increase in liking or umami intensity but in terms of whether mushroom incorporation helps narrow specific sensory differences between plant-based formulations and their animal-derived counterparts.

### 5.1. Instrumental Approaches for Predicting Sensory Quality: Integrating Electronic Sensors with Human Evaluation

Human sensory evaluation remains essential for determining whether a formulation change produces a perceptible and desirable difference. Instrumental methods, however, can provide complementary information on the chemical and physicochemical changes underlying these responses [41,43]. Gas chromatography–mass spectrometry (GC–MS) can characterize individual volatile compounds, whereas electronic noses and electronic tongues generate multidimensional response patterns that can support rapid discrimination and formulation screening [44,45].

The complementary value of these approaches is illustrated by a study comparing three commercial plant-based beef analogues with beef using HS-SPME-GC–MS, an electronic nose, an electronic tongue, and sensory evaluation. A total of 126 volatile compounds were identified across the plant-based products and beef, while the instrumental and sensory measurements revealed substantial differences in aroma and taste profiles after thermal processing [43]. The study therefore illustrates why chemical profiling alone is insufficient to establish sensory equivalence: differences in volatile composition were accompanied by differences in perceived aroma and taste.

For mushroom-derived ingredients, the greatest value of instrumental approaches therefore lies in linking chemical fingerprints with sensory outcomes rather than replacing human evaluation [40,41]. This is particularly relevant because mushroom incorporation can simultaneously alter desirable attributes, such as savoury and meat-like aroma, and undesirable attributes, such as bitterness, astringency, or characteristic mushroom notes. In oyster-mushroom mycelium-fortified plant-based beef analogues, for example, moderate mycelium incorporation increased selected volatile compounds and meat-like and fat-like aroma while reducing beany flavour; sensory and electronic-tongue measurements also indicated reduced bitterness and astringency at mycelium levels below 8% [40]. Such combined analytical–sensory strategies can therefore identify useful formulation windows while avoiding the assumption that a stronger instrumental signal necessarily corresponds to greater consumer acceptance.

### 5.2. Masking Off-Flavours to Improve Consumer Acceptance

Flavour optimisation in plant-based foods should not be reduced to increasing umami intensity. “Beany, grassy, bitter, and other undesirable notes can remain important sensory limitations, meaning that reducing their perception is as relevant as enhancing desirable attributes…” [8,42]. The objective is therefore to achieve a more balanced flavour profile rather than to maximise any individual taste attribute.

Mushroom-derived materials may contribute to this process through simultaneous effects on taste and aroma. In plant-based beef analogues, incorporation of oyster-mushroom mycelium was associated with reduced beany flavour, bitterness, and astringency while increasing meat-like and fat-like aroma at moderate incorporation levels [40]. These findings are important because they indicate that the sensory contribution of mushroom materials may arise from rebalancing the relative prominence of desirable and undesirable cues, rather than from simply increasing glutamate-driven umami intensity [40].

The masking effect remains strongly dependent on formulation conditions. Food-matrix composition and processing can alter the release and perception of volatile and taste-active compounds [17,46], while incorporation level can determine whether mushroom-derived materials improve or compromise sensory quality [40]. Consequently, successful application requires identification of an optimal inclusion range in which desirable savoury and aromatic attributes are strengthened without generating new sensory limitations [47].

Recent formulation studies indicate that the sensory performance of edible mushroom-derived ingredients depends on multiple interacting factors rather than on ingredient composition alone. Representative examples illustrating these relationships across different plant-based food systems are summarized in Table 2.

Across these studies, sensory effects were dependent on mushroom species, material type, incorporation level, and food matrix. The available evidence therefore supports the potential of mushroom-derived materials to improve flavour balance, but it does not justify treating all mushroom-derived materials as interchangeable flavour enhancers. Direct comparisons remain difficult because studies differ in ingredient preparation, formulation, sensory methodology, and the extent to which consumer acceptance was assessed.

## 6. Industrial Translation: Opportunities and Challenges

The scientific rationale for incorporating mushroom-derived ingredients into plant-based foods is becoming increasingly convincing, but translating this potential into commercial applications requires more than demonstrating sensory activity under experimental conditions [11]. Industrial adoption depends on reproducible raw materials, stable functionality, processing compatibility, regulatory compliance, safety, consumer acceptance, and economic feasibility [10,48,49]. Mushroom by-products are particularly attractive within this context because their recovery can reduce material losses while providing food ingredients with nutritional, functional, and flavour-related properties [11,49]. Their industrial value therefore lies not only in replacing conventional flavour enhancers, but also in combining flavour functionality with resource valorisation.

Batch-to-batch variability represents one of the main barriers to this transition. Mushroom by-products are not chemically uniform materials; their composition can vary with species, tissue fraction, cultivation conditions, substrate, and subsequent processing. These factors can affect the concentration of bioactive and flavour-active constituents and, consequently, the functional performance of the resulting ingredient [11,14]. Processing introduces an additional source of variability because extraction, hydrolysis, drying, and other treatments can alter both composition and functionality [10]. Industrial development should therefore begin with defined raw-material specifications and controlled processing conditions rather than treating different mushroom by-products as interchangeable flavour sources.

Food safety and storage stability represent additional constraints that must be addressed before mushroom by-products can be used as food ingredients at scale. Fresh residual mushroom biomass is highly perishable because of its high moisture content, making timely stabilisation, appropriate drying, and controlled storage important for limiting quality deterioration before further processing [11]. In addition to microbiological quality, safety assessment should consider the possible presence or accumulation of chemical contaminants, including heavy metals and other undesirable substances, depending on the mushroom species, cultivation substrate, environmental conditions, and processing history [11].

More broadly, the valorisation of food by-products requires systematic physicochemical and microbiological assessment because residual streams may carry hazards that are not adequately controlled by their subsequent use as ingredients [50]. These considerations highlight the need for defined raw-material specifications covering identity, moisture content, microbiological quality, contaminant limits, and compositional characteristics. Such standardisation is essential for ensuring that variability among batches does not compromise the safety, stability, or sensory functionality of mushroom-derived ingredients [10].

Economic feasibility presents a further challenge. The production of a standardised mushroom-derived flavour ingredient may require additional operations such as stabilisation, drying, extraction, hydrolysis, storage, and quality control, and these steps can determine whether valorisation is economically competitive with alternative ingredients [10]. Consequently, technological feasibility should be evaluated together with processing yield, energy and enzyme requirements, ingredient stability, and the cost of achieving a consistent sensory specification. This is particularly important because laboratory-scale extraction or formulation studies do not necessarily demonstrate economic viability at industrial scale [49].

From this perspective, the strongest industrial opportunity does not lie simply in replacing conventional flavour enhancers. Rather, it lies in developing standardised, multifunctional ingredients that can improve flavour balance while simultaneously valorising underutilised mushroom biomass [49]. Such an approach may support the development of more acceptable plant-based foods by addressing sensory limitations while contributing to the more efficient use of underutilised mushroom biomass [48]. Achieving this transition will require integration of raw-material standardisation, controlled processing, instrumental–sensory prediction, consumer validation, safety assessment, and techno-economic evaluation. Future research should therefore move beyond proof-of-concept studies toward multi-batch validation in representative food matrices and pilot-scale production, with predefined specifications for composition, sensory performance, safety, stability, and cost. This approach would provide a more realistic pathway from mushroom-by-product valorisation to commercially viable flavour-enhancing ingredients [49]. When appropriately formulated, such products may also contribute to health-oriented dietary patterns, although their nutritional value ultimately depends on the composition and processing of the complete food matrix [3].

## 7. Conclusions

Current evidence indicates that edible mushroom by-products have potential as multifunctional ingredients for improving the sensory quality of plant-based foods. Their contribution is not determined by glutamate concentration alone but by interactions among taste-active compounds, aroma constituents, food-matrix characteristics, processing conditions, and sensory perception. Evidence from plant-based food applications suggests that mushroom-derived materials can enhance savoury and aromatic attributes while attenuating undesirable notes such as beany, bitter, or astringent perceptions. However, these effects remain dependent on mushroom source, processing history, incorporation level, and food matrix.

The sustainability value of this approach lies in converting underutilised mushroom biomass into value-added food ingredients, but by-product utilisation should not automatically be equated with environmental sustainability. Processing requirements, energy and resource consumption, ingredient stability, safety, economic feasibility, and the environmental performance of the resulting food system should be considered when evaluating the overall sustainability of this strategy. Similarly, potential nutritional and health benefits should be assessed for the specific mushroom-derived ingredient and final food rather than inferred solely from the use of mushroom biomass.

Several limitations currently restrict broader translation. The available literature is heterogeneous with respect to mushroom species, by-product definitions, processing histories, ingredient concentrations, food matrices, and sensory methodologies. Evidence is also concentrated in laboratory and model-food systems, whereas multi-batch validation, consumer studies, long-term stability assessment, and pilot- or industrial-scale trials remain comparatively limited. These limitations currently constrain direct comparison among studies and the development of universal formulation guidelines.

Future research should therefore prioritise standardised characterisation and processing of mushroom by-products, followed by integrated assessment of flavour-active composition, instrumental responses, sensory performance, and consumer acceptance in representative plant-based food matrices. Multi-batch and pilot-scale investigations should additionally incorporate safety, regulatory, economic, and environmental assessments. Such an integrated perspective may provide a more robust scientific basis for developing plant-based foods that combine improved sensory quality with more sustainable use of underutilised mushroom biomass, while recognizing that potential health benefits depend on the nutritional quality of the final food formulation.

## Figures and Tables

**Table 2:** Reported sensory effects of mushroom-derived materials in plant-based food systems and their relevance to by-product valorisation.

Mushroom-Derived Material	Material Origin/By-Product Status	Plant-Based Food Matrix	Primary Sensory Effect	Consumer/Sensory Response	Proposed Contribution	Key Limitation
*Agaricus bisporus* trimmings/powder	Mushroom-derived material; by-product status not established in the cited study	Plant-based beef burger/meat analogue	Improved tenderness and moisture-related sensory attributes at moderate mushroom substitution levels	Consumer acceptance remained comparable to the control up to 15% mushroom substitution	Contribution of mushroom-derived taste and aroma compounds, together with matrix-dependent sensory effects	Responses varied with incorporation level and formulation [39]
*Pleurotus ostreatus mycelium*	Mushroom-derived material; by-product status not established in the cited study	Plant-based meat alternative/beef analogue	Enhancement of savoury continuity and flavour complexity; reduction in beany flavour, bitterness, and astringency	More favourable sensory profile at moderate incorporation levels	Combined modification of volatile and non-volatile flavour characteristics	Excessive incorporation may alter characteristic mushroom notes; optimum level is formulation-dependent [40]
*Lentinula edodes* mushroom powder	Mushroom-derived material; not demonstrated as a residual by-product stream	Pea–mung bean composite protein	Modification of aroma and flavour profile, with reduction in selected off-flavour-related volatile compounds	Flavour quality was optimised at an intermediate incorporation level, with 20% identified as the preferred level	Modification of the flavour profile through changes in volatile compounds and matrix interactions	The study used mushroom powder rather than a demonstrably residual by-product stream, limiting direct extrapolation to mushroom-residue valorisation [47]
*Schizophyllum commune* extract/powder	Mushroom-derived processed ingredient; by-product status not established	Reduced-sodium soup/model system	Umami–salt synergism	Improved savoury perception under reduced-sodium conditions	Interaction of mushroom-derived taste-active constituents with salt and other taste-active components	Evidence derived mainly from model/reduced-sodium systems rather than commercial products [15]

Note: For consistency with the scope of this review, a mushroom material is classified as a by-product only when its residual origin is explicitly established in the cited source. Materials for which such an origin is not demonstrated are retained as mushroom-derived ingredients/materials and are not treated as direct evidence of mushroom by-product valorisation.

## Data Availability

No new data were created or analyzed in this study. Data sharing is not applicable to this article.
